# Properties of Iron Bacteria Biofouling on Ni-P-rGO Coating under Flowing Conditions

**DOI:** 10.3390/ma13030764

**Published:** 2020-02-07

**Authors:** Mingyang Sun, Zhiming Xu, Zuodong Liu, Bingbing Wang, Huishuang Di

**Affiliations:** School of Energy and Power Engineering, Northeast Electric Power University, Jilin 132012, China; sunmingyang0313@163.com (M.S.); liuzuodong@neepu.edu.cn (Z.L.); wangbb@neepu.edu.cn (B.W.); dhs_0411@163.com (H.D.)

**Keywords:** biofouling, iron bacteria, nickel-phosphorus-reduced graphene oxide (Ni-P-rGO), induction period, fouling resistance

## Abstract

Biofouling on heat exchange devices can decrease heat transfer efficiency, corrode materials, and even lead safety accidents. Most heat exchange devices are made of carbon steel, which produces biofouling easily. In this paper, nickel-phosphorus-reduced graphene oxide (Ni-P-rGO) coating was prepared on carbon steel by electroless plating as a kind of advanced material to study the properties of iron bacteria biofouling under flowing conditions. The coating was analyzed via scanning electron microscopy and Raman spectroscopy. The properties of iron bacteria biofouling on carbon steel and Ni-P-rGO coating were then compared under flowing conditions. Compared with carbon steel, the asymptotic value of fouling resistance on the Ni-P-rGO coating significantly decreased. Additionally, the induction period and the time of reaching the asymptotic value greatly increased. The inhibition properties of biofouling of advanced materials Ni-P-rGO coating under different temperatures, flow velocities, and initial concentrations was also studied.

## 1. Introduction

Biofouling is a biofilm or an organic film that is formed by bacteria, algae, and other microorganisms, and their excreta deposited on a solid surface [1]. Biofouling may not only decrease the heat transfer efficiency but also corrode the materials and even lead to accidents [2]. Xu et al. [3] studied the characteristics of iron bacteria and slime-forming bacteria biofouling in plate heat exchangers. Trueba et al. [4] investigated the effect of biofilms on the heat transfer process in the seawater cooled stainless steel condenser surface. The results showed that when the biofilm was thinner, the heat transfer process was better. Chen et al. [5] explored the growth characteristics of microorganisms on the surface of the heat exchanger under the conditions of changing temperature, flow velocities, and microbial concentration.

In the past 20 years, modified surfaces have been widely used in various industries due to the properties of anti-fouling, anti-corrosion, and non-pollution. Additionally, there have been several reports on the modified surfaces to inhibit the biofouling. Shao et al. [6] found that the silver coating can inhibit the attachment of *Pseudomonas aeruginosa (PA01)*. Xu et al. [7] demonstrated that electroless Ni-Cu-P coating has excellent anti-biofouling properties. Liu and Zhao. [8] found that the coating of Ni-P-ploy tetra fluoro ethylene (PTFE) exhibits excellent antimicrobial properties and reduced the *Escherichia (XA90)* adhesion by 95% as compared to stainless steel 304.

Because carbon materials have good superior chemical stability and high surface area [9], several researchers have added carbon materials to the electroless plating solution of Ni-P coating, and the resultant coating has good wear resistance, corrosion resistance, and antifouling. Tran et al. [10] produce high-performance CNT (carbon nano tube) /Au/Cu composite wires via sputtering and electroplating. At the CNT volume fraction of more than 20%, the composite wires were lightweight and possessed the combined properties of effective strength and high electrical conductivity. Wu et al. [11] showed that the Ni-P-graphene oxide (GO) coating on the mild steel surface possesses the best anti-wear and microhardness properties when 40 mg/L graphene oxide is added into the electroless plating solution. Qian et al [12] found that the G-Ni-P composite plating increased the Vickers hardness and Young’s modulus as compared with the Ni-P coating. Matjie et al. [13] prepared a modified diamond-like carbon (DLC) coating that reduced the adhesion of aluminum silicate deposit by 97% as compared with uncoated stainless steel. Shao et al. [14] added graphene oxide to reinforce the Ni-P coating material in order to inhibit the adhesion of *Staphylococcus aureus (ATCC 6538)*. Lee et al. [15] prepared the Ni-P/diamond, Ni-P/graphene, and Ni-P/diamond/graphene coating, and the Ni-P/diamond/graphene coating displayed the best hardness, corrosion resistance, and wear resistance in 3.5 wt % NaCl solution.

Although there has been significant work on biofouling and modified surfaces, there have been limited reports about the properties of biofouling on the nickel-phosphorus-reduced graphene oxide (Ni-P-rGO) coatings. The experimental conditions of the modified surface to inhibit fouling was almost static, as were the experimental conditions under flow conditions. Herein, Ni-P-rGO coating was prepared by electroless plating on carbon steel, and the iron bacteria was used as the scaling bacteria to study the biofouling properties of iron bacteria on Ni-P-rGO coating under flow conditions.

## 2. Experimental

### 2.1. Experimental Setup

**Figure 1:**
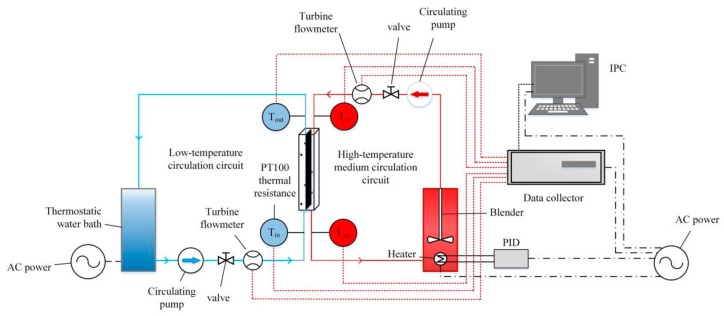
Schematic of experimental setup.

Figure 1 shows the schematic of the experimental setup, where the solid line is the flow channel and the dotted line is the connection line of the data acquisition system. The experimental setup consisted of a low-temperature medium circulation circuit, a high-temperature medium circulation circuit, an experimental section, and a data acquisition system. During the experiment, the high temperature work fluid and low temperature work fluid exchanged heat in the experimental section via circulating pumps and flow meters, and their inlet and outlet temperatures were recorded through the data acquisition system.

### 2.2. Experimental Principle and Method

The heat absorption by the low temperature working fluid during operation is equal to the heat release by the high temperature working fluid [3]:

(1) $$\phi_{1}=\phi_{2}\;q_{m1}c_{p1}\left(t_{1}^{\prime \prime }-t_{1}^{\prime }\right)=q_{m2}c_{p2}\left(t_{2}^{\prime }-t_{2}^{\prime \prime }\right)$$

where $\phi_{1}$ is the heat release by the high temperature working fluid in W; $\phi_{2}$ is the heat absorption by the low temperature working fluid in W; $c_{p1}\;$and $c_{p2}\;$are the specific heat of high temperature working fluid and low temperature working fluid, respectively, at constant pressure in kJ (kg K)^−1^; $q_{m1}\;$and $q_{m2}$ are the mass fluid velocity of the high temperature working fluid and low temperature working fluid in kg s^−1^, respectively; and $t_{1}^{\prime }$, $t_{1}^{\prime \prime }$, $t_{2}^{\prime }$, and $t_{2}^{\prime \prime }$ are the inlet and outlet temperatures of the high temperature working fluid and low temperature working fluid in K.

Hence, the heat transfer coefficient can be calculated by:

(2) $$k=\frac{\phi }{A\Delta t_{m}}=\frac{q_{m1}c_{p1}\left(t_{1}^{\prime \prime }-t_{1}^{\prime }\right)+q_{m2}c_{p2}\left(t_{2}^{\prime }-t_{2}^{\prime \prime }\right)}{2A\Delta t_{m}}\;\Delta t_{m}=\frac{\left(t_{2}^{\prime \prime }-t_{1}^{\prime }\right)-\left(t_{2}^{\prime }-t_{1}^{\prime \prime }\right)}{ln\frac{t_{2}^{\prime \prime }-t_{1}^{\prime }}{t_{2}^{\prime }-t_{1}^{\prime \prime }}}$$

The fouling resistance *R_f_* is defined as:

(3) $$R_{f}=\frac{1}{k}-\frac{1}{k_{0}}$$

where *A* is the heat exchange surface in m^2^; $\Delta t_{m}\;$is the logarithmic mean temperature difference in K; and *k_0_* and *k* are the overall heat transfer coefficient of clean (unfouled) surfaces and fouled surfaces in W (m^2^ K)^−1^.

### 2.3. Stability Verification of the Experimental Setup

**Figure 2:**
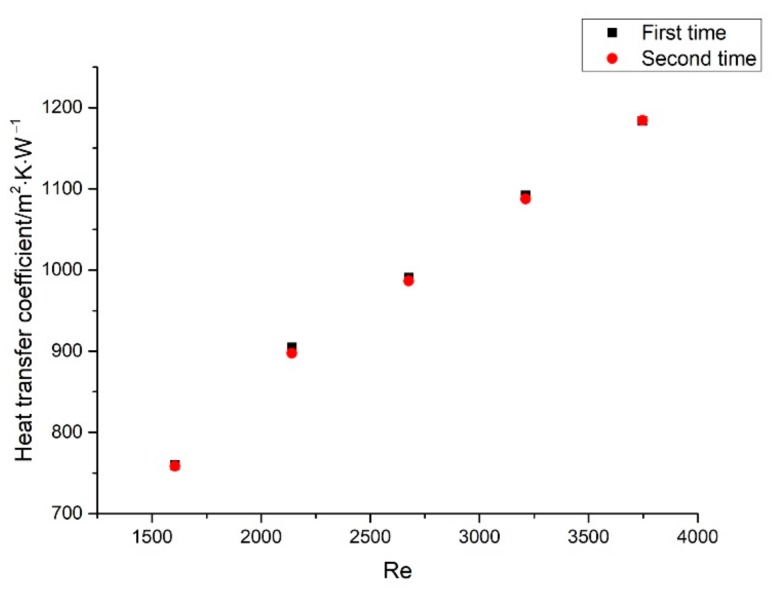
Heat transfer coefficients of the experiment setup at different flow velocities.

In order to verify the stability of the experimental setup, repeatability experiments were conducted. The experiments were performed by taking distilled water as the circulating working fluid, high temperature working fluid at 65 °C, and 0.35 m/s and low temperature working fluid at 30 °C, increasing the flow velocity of the low temperature working fluid from 0.15 m/s to 0.35 m/s. The overall heat transfer coefficients for the repeatability experiments are shown in Figure 2. Under the conditions of changing the flow velocity, the maximum difference in the heat transfer coefficient of the two experiments was lower than 1%, which verifies the stability of the experimental setup.

### 2.4. Coating Preparation and Iron Bacteria Culture

**Figure 3:**
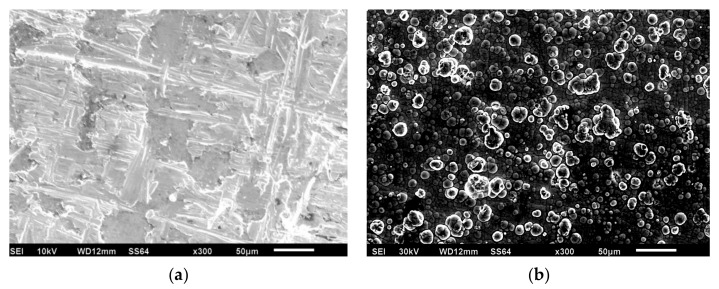

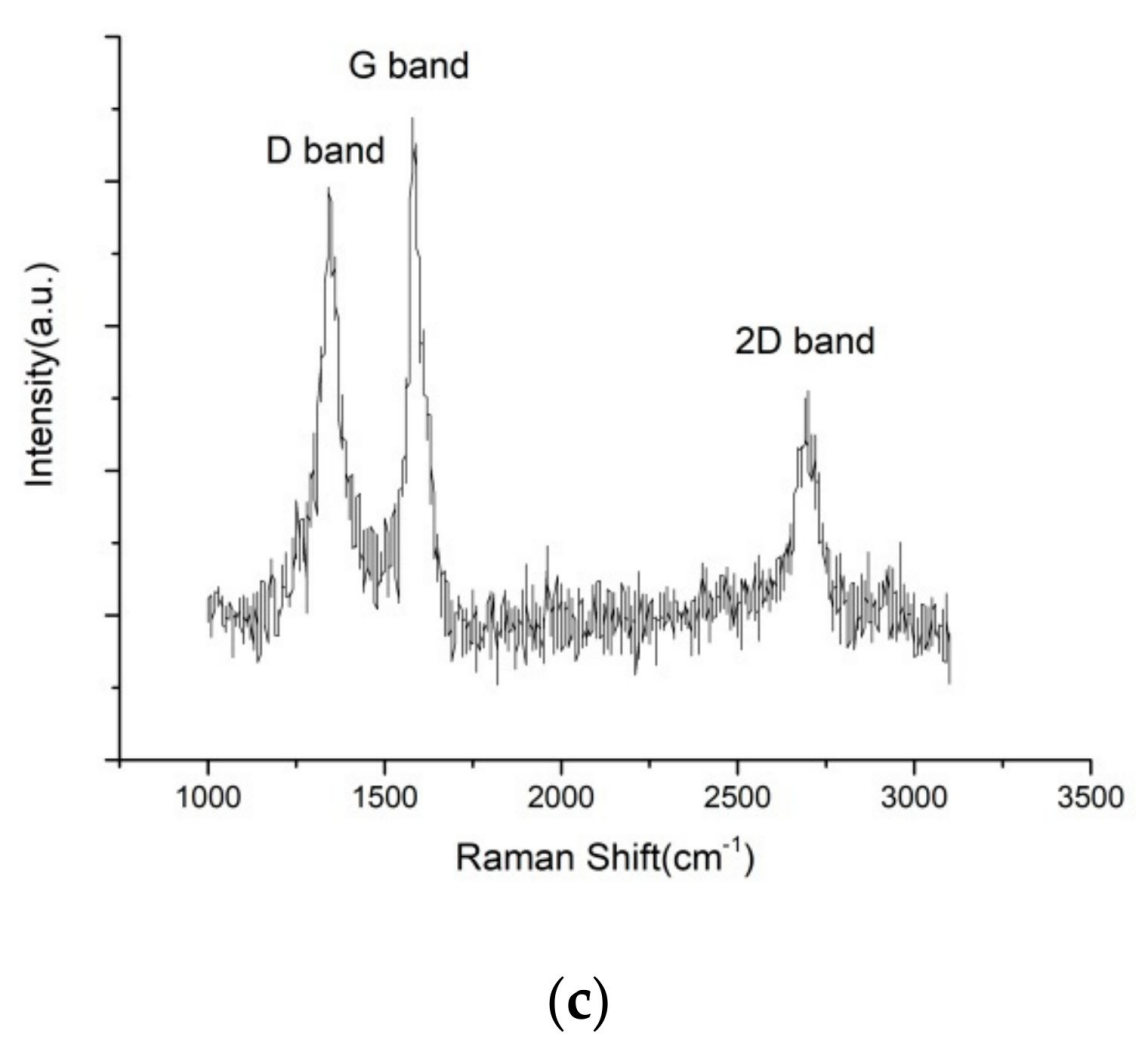
SEM morphology of carbon steel (**a**), nickel-phosphorus-reduced graphene oxide (Ni-P-rGO) coating (**b**), and Raman spectrum of rGO in Ni-P-rGO coatings (**c**).

The Ni-P-rGO coating material was prepared on Q235 carbon steel (500 mm × 50 mm × 0.5 mm) via electroless plating. Before electroless plating, the substrates were first polished with an 800-grit waterproof abrasive paper and a 600-grit waterproof abrasive paper to make the surface smooth and flat, respectively. The oil was then removed using alkali washing and the rust was removed using 20% sulfuric acid for 1 min. Finally, the substrates were placed into 10% sulfuric acid for 1 min to activate the surface. After each step, the carbon steel surface was cleaned with distilled water. Table 1 lists the electroless bath formulation composition and operating conditions. The pH of the electroless bath solution was adjusted to 4.8 by adding ammonium hydroxide. The electroless plating process lasted 120 min. As shown in Figure 3, after successful plating, the coating was analyzed via scanning electron microscope and Raman spectroscopy. Figure 3a,b displays the morphology of carbon steel and Ni-P-rGO coating under a scanning electron microscope. The surface of the carbon steel was uneven and had several scratches and the Ni-P-rGO coating showed greater flatness than carbon steel in the microstructure. However, there were several inlaid nodules in the Ni-P-rGO coating, confirming the presence of graphene with a high specific surface area to stimulate the nucleation [13]. Figure 3c shows the Raman spectrum of the Ni-P-rGO coating. There were three evident peaks at 1350 cm^−1^, 1580 cm^−1^, and 2700 cm^−1^, which correspond to the D band, G band, and 2D band. The D band is assigned to the breathing mode of the ĸ-point phonons with A_1g_ symmetry [16] and it associates with the edge defects of graphene. The G band introduces the E_2g_ phonon of the carbon sp^2^ atoms, which is due to the highly ordered graphite [17]. The 2D band was generated by double phonon double resonance, which is closely related to the band structure of graphene [18].

The iron bacteria used in this study was isolated and purified from the slime of the cooling tower in the power plant. The composition of liquid medium for iron bacteria is listed in Table 2. Before inoculating the iron bacteria, the pH of medium was adjusted to 6.8 and sterilized using autoclave for 30 min. After inoculation, the medium was placed into an incubator for 72 h at 30 °C.

### 2.5. Experimental Progress

Before adding the iron bacteria into the thermostatic water bath, adjustment of the operation conditions was necessary. The fluid velocity was adjusted by the flow regulating valve and monitored via the flowmeter. The hot water was heated by the electric heater and controlled by the temperature controller. When the experimental conditions were stable, the iron bacteria was added into the thermostatic water bath. The data acquisition system collected data four times per minute. The collected data, such as inlet and outlet temperatures and flow velocity, were transmitted to the computer for storage. The data were processed after finishing the experiment. During the experiment, the concentration of iron bacteria in water was measured every 12 h using a spectrophotometer.

## 3. Results and Discussions

### 3.1. Comparison of Properties of Iron Bacteria Biofouling between Carbon Steel and Ni-P-rGO Coating

**Figure 4:**
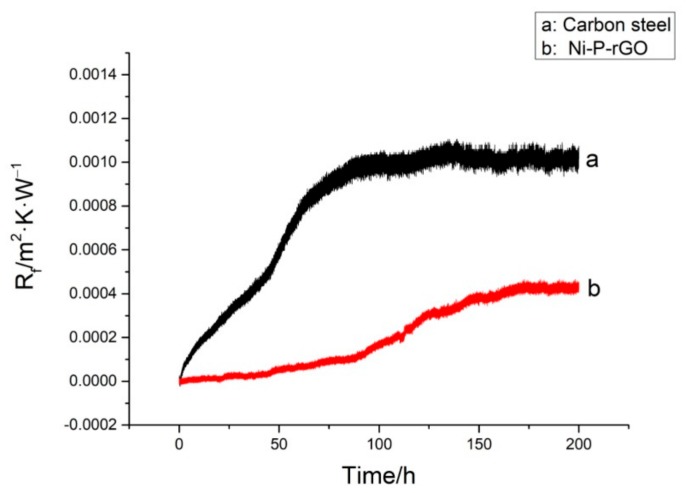
The properties of the iron bacteria biofouling between carbon steel and Ni-P-rGO coating.

**Figure 5:**
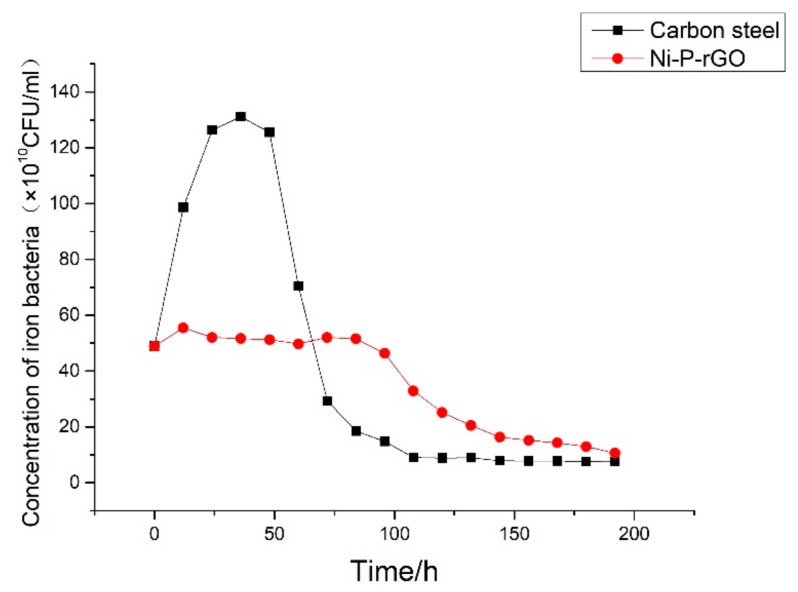
Variation of iron bacteria concentration during the experiment in the low temperature working fluid.

**Figure 6:**
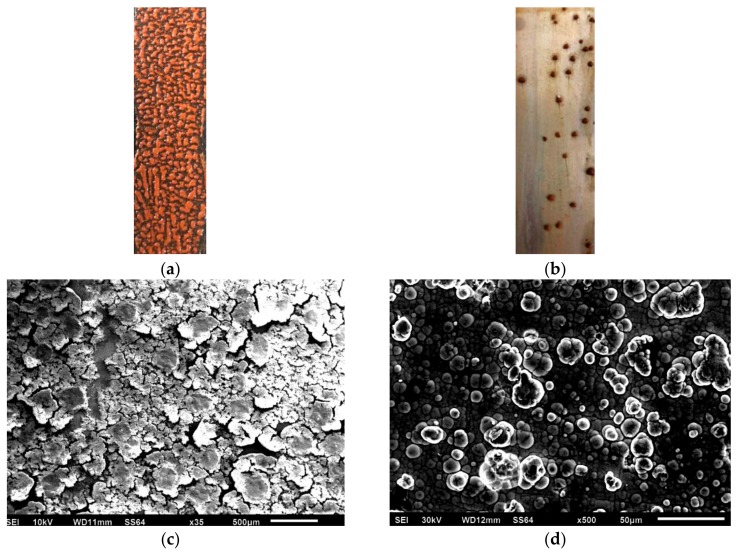
Macrograph of carbon steel surface (**a**) and Ni-P-rGO coating (**b**) after experiment; micrograph surface of carbon steel (**c**) and Ni-P-rGO coating (**d**) after experiment.

The experiments of carbon steel and Ni-P-rGO coating were performed when the conditions were with a low temperature working fluid at 30 °C, flow velocity at 0.25 m/s, and initial concentration of iron bacteria at 48.91 × 10^10^ CFU/mL, and with the high temperature working fluid at 65 °C, the flow velocity was at 0.35 m/s. The results are shown in Figure 4. As shown in the figure, the asymptotic value of the fouling resistance of Ni-P-rGO coating was much smaller than the carbon steel. Moreover, the fouling resistance of carbon steel increased rapidly as compared with the Ni-P-rGO coating. There was no induction period on carbon steel and it only took 90 h to reach the asymptotic value of fouling resistance. However, the Ni-P-rGO coating had an induction period of about 40 h, and the rate of fouling growth was extremely slow and took 175 h to reach the asymptotic value of fouling thermal resistance. This period of time was almost twice as long as the carbon steel. The changes in the concentration of iron bacteria during the experiment are shown in Figure 5. The concentration of iron bacteria in the carbon steel experiment increased significantly at the initial stage and decreased rapidly after reaching the peak at 36 h. However, at the initial stage of the Ni-P-rGO coating experiment, the concentration of iron bacteria almost remained invariable and decreased rapidly after 80 h. This phenomenon can be explained by the theory that the iron element in carbon steel provides for the growth and reproduction of iron bacteria. At the beginning of the experiment, the iron bacteria greatly multiplied and adhered to the surface of carbon steel and the carbon steel was corroded to produce a large number of corrosion products. When iron bacteria multiplied too much, the surface of carbon steel was gradually covered by iron bacteria biofouling and the iron bacteria could not contact the carbon steel to obtain nutrients. At the same time, large amounts of iron bacteria also consumed large amounts of nutrients. Therefore, bacteria decayed rapidly and eventually reached an asymptotic value of fouling resistance. In contrast, the iron bacteria on the coating could not obtain any nutrients and made contact with graphene in the coating during the corrosion process. Graphene has a special hexagonal structure that can puncture the cytomembrane of iron bacteria [19]. The propagating iron bacteria and the decaying iron bacteria reached a dynamic balance; hence, the concentration of iron bacteria was almost invariable at the initial stage of the experiment. After a period of corrosion by the iron bacteria, increasingly more graphene was exposed and there were less nutrients in the water; therefore, the iron bacteria decayed rapidly and reached an asymptotic value of fouling resistance. Figure 6 shows the macrograph and micrograph of the carbon steel and coating surface after the experiment. As the macrograph shows, the surface of carbon steel was almost covered with red-brown iron bacterial fouling. However, the surface of the Ni-P-rGO coating had only sporadic iron bacteria biofouling. As the micrograph shows, there was not only a thicker layer of biofouling, but also a significant iron bacteria colony on the carbon steel. However, on the coating of the Ni-P-rGO, biofouling was almost absent and the coating surface was almost free of corrosion damage.

### 3.2. Effect of Temperature on the Fouling Characteristics of Iron Bacteria on Ni-P-rGO Coating

**Figure 7:**
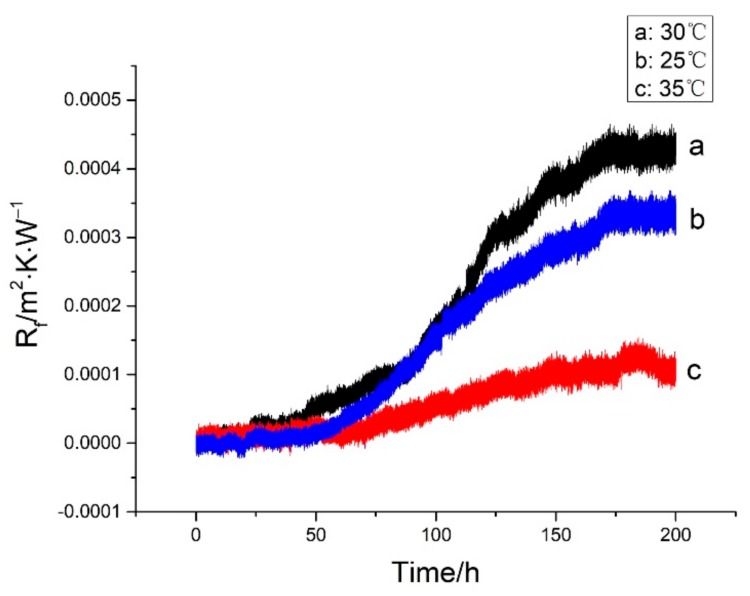
Effect of temperature on iron bacteria biofouling characteristics on the Ni-P-rGO coating.

**Figure 8:**
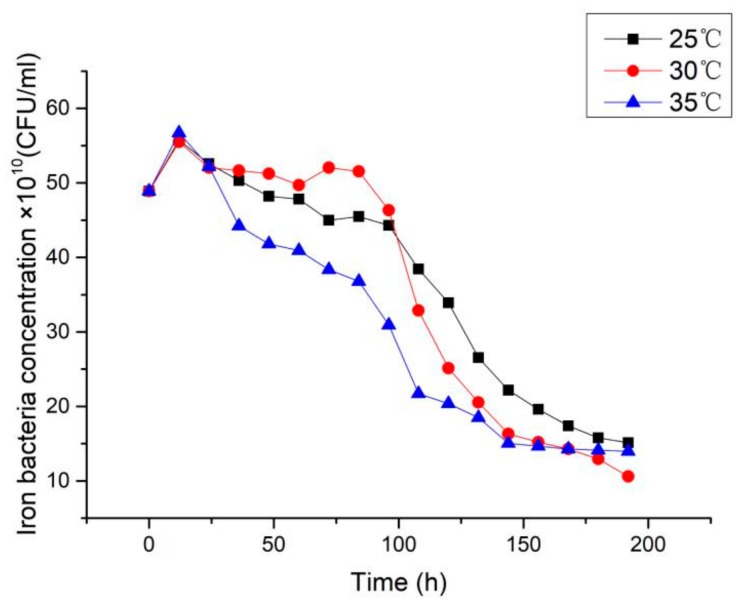
Variation of iron bacteria concentration at different temperatures in the low temperature working fluid.

By maintaining other operating conditions, three groups of comparative experiments were performed by changing the temperature of the low temperature working fluid. The effect of the temperature on the biofouling characteristics of iron bacteria for three different temperatures is shown in Figure 7. As shown, the induction period existed in all the three experimental temperatures. When the temperature was 30 °C, the asymptotic value of the fouling resistance was the largest, while the induction period was the shortest. When the temperature was 35 °C, the asymptotic value of the fouling resistance was the smallest, while the induction period was the longest. When the temperature was 25 °C, the asymptotic value of the fouling resistance and the induction period were between the other two experiments. Figure 8 shows the variation of concentration of iron bacteria at different temperatures during the experiment. At 25 °C and 35 °C, the concentration of iron bacteria was almost always below that at 30 °C. With the increase in temperature, the Brownian motion was more intense, leading to an increase in the probability for iron bacteria to impact and adhere to the surface. Therefore, the time to reach the asymptotic value of fouling resistance was shortened at 35 °C. However, the activities of enzymes can also affect the reproduction and growth of bacteria [20]. At 30 °C, enzymes of the iron bacteria had more optimal activities than those at 25 °C and 35 °C. The iron bacteria with higher activity had a higher opportunity to adhere to the surface. Therefore, the induction period was the shortest and the asymptotic value of fouling resistance was the highest at 30 °C. In the initial stage of experiment, the number of iron bacteria at 30 °C was much more than those at 25 °C and 35 °C. But the iron bacteria reproduced and grew rapidly at 30 °C, and the coating corrosion was relatively serious. This led to increased exposed graphene and a fast consumption of the nutrients in the water. Hence, the concentration of iron bacteria declined rapidly after reaching a peak.

### 3.3. Effect of Flow Velocity on Fouling Characteristics of Iron Bacteria on Ni-P-rGO Coating

**Figure 9:**
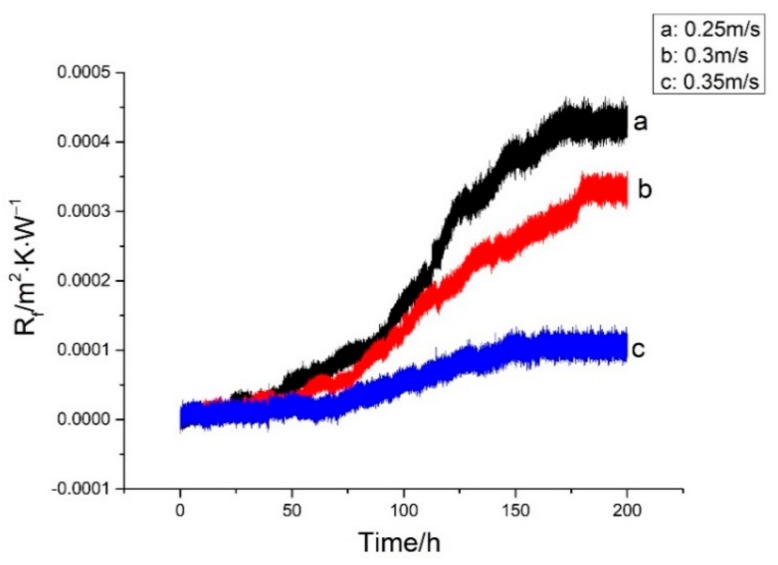
Effect of flow velocity on iron bacteria biofouling characteristics on Ni-P-rGO coating.

**Figure 10:**
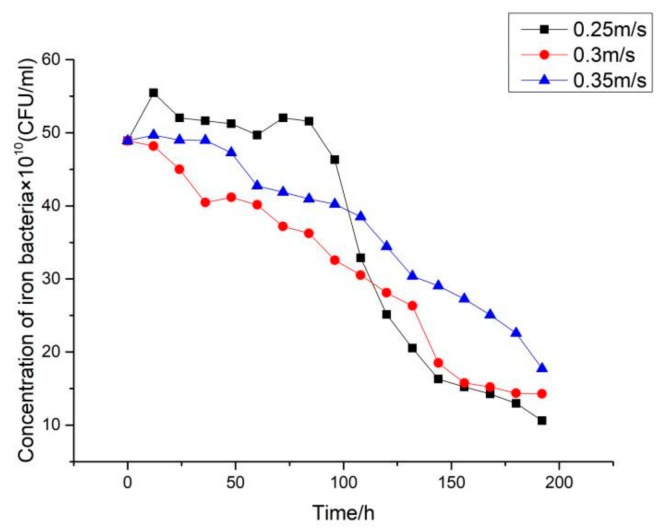
Variation of iron bacteria concentration at different flow velocities in the low temperature working fluid.

By keeping other operating conditions unchanged, three groups of comparative experiments were performed by changing the flow velocity of the low temperature working fluid. The effect on characteristics of iron bacteria biofouling due to three different flow velocities is shown in Figure 9. As shown in the figure, with the increase in flow velocity, the induction period increased and the asymptotic value of fouling resistance decreased. In the fouling deposition stage, the growth and detachment of biofilm are simultaneous [3]. The accelerated flow velocity increased the shear force of the fluid on the coating surface, and the higher shear force peeled more growth biofilm. Therefore, the induction period increased with the increase in flow velocity. Furthermore, as the flow velocity increased, the thinner biofilm attached to the surface, therefore, the solid content was lower [4], and the asymptotic value of the fouling resistance decreased. However, higher flow velocity provided more oxygen and nutrients, which led to the shortening of time to reach the asymptotic value of fouling resistance at 0.35 m/s. Figure 10 shows the variation of iron bacteria concentration at different flow velocities during the experiment. As shown in Figure 10, the concentration of iron bacteria kept decreasing at 0.3 m/s and 0.35 m/s, but raised first and then declined at 0.25 m/s. Though higher velocity provided more oxygen and nutrients, the iron bacteria came into contact with graphene more frequently. Therefore, at 0.25 m/s, the reproduced and decayed iron bacteria reached a dynamic balance at the initial stage of the experiment. At 0.3 m/s and 0.35 m/s, decayed iron bacteria were more than the reproduced iron bacteria.

### 3.4. Effect of Concentration on the Biofouling Characteristics of Iron Bacteria on Ni-P-rGO Coating

**Figure 11:**
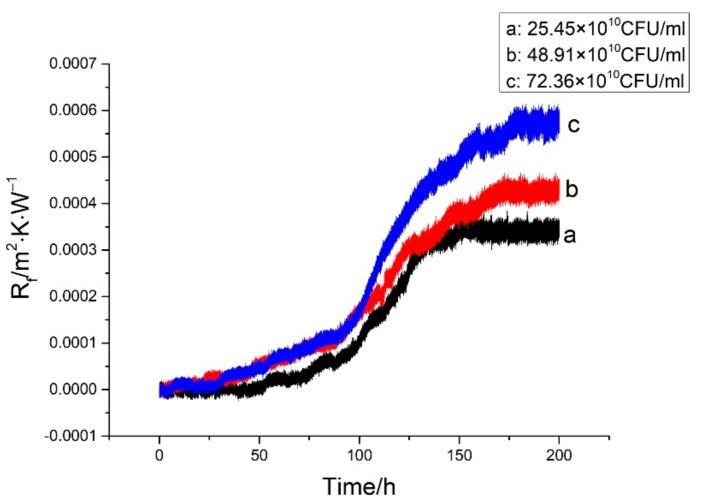
Effect of concentration on iron bacteria biofouling characteristics on Ni-P-rGO coating.

**Figure 12:**
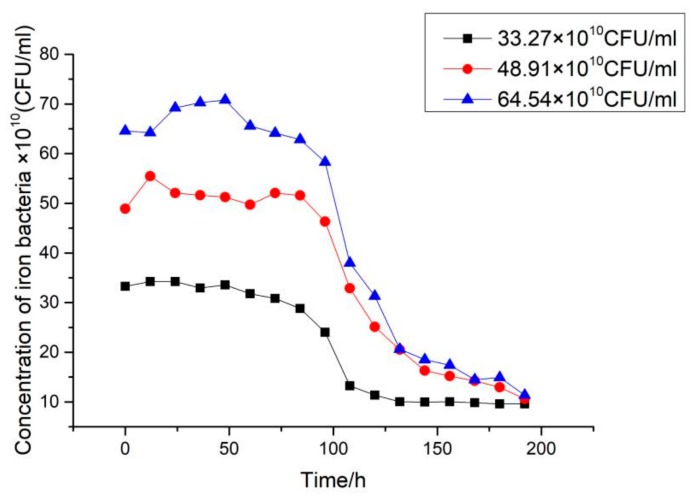
Variation of iron bacteria concentration at different initial concentrations in the low temperature working fluid.

By keeping other operating conditions unchanged, three groups of comparative experiments were conducted by changing the concentration of iron bacteria in the low temperature working fluid. The effect on the characteristics of iron bacteria biofouling for three different concentrations is shown in Figure 11. As shown in the figure, with the increase in iron bacteria concentration, the induction period decreased, while the asymptotic value of fouling thermal resistance increased. The initial conditions for the formation of the biofouling is the formation and adhesion of the biofilm, and the formation of the biofilm is related to the absorption of protein molecules and organic polymer [21]. As the concentration of iron bacteria increased, more protein molecules and organic polymers were secreted and the biofilm was formed quickly. Therefore, the induction period and time to reach the asymptotic value of fouling resistance were shortened. Furthermore, the asymptotic value of fouling resistance was increased. Figure 12 shows the variation in the different concentrations of iron bacteria during the experiments. At high concentrations of iron bacteria, there was more dramatic decay of iron bacteria. The reason for the phenomenon is that the increase in iron bacteria concentration led to more iron bacteria reproduction. However, when the nutrients in the water were depleted, the iron bacteria decayed dramatically.

## 4. Conclusions

[1] Compared to the carbon steel, which was used to prepare the heat exchange device as raw materials, the advanced materials with Ni-P-rGO coating had an important property that inhibited biofouling. They not only reduced the asymptotic value of the fouling resistance of iron bacteria fouling significantly, but also greatly increased the induction period and fouling growth time.
[2] The inhibition of advanced materials with Ni-P-rGO coating against iron bacteria biofouling at different temperatures was studied. The asymptotic value of fouling resistance was highest at 30 °C, and the induction period was the shortest at this temperature. The asymptotic value of fouling thermal resistance was lowest at 35 °C, and the induction period was longest at this temperature. The asymptotic value of fouling resistance and the induction period were between the former two at 25 °C.
[3] The inhibition of advanced materials with Ni-P-rGO coating against iron bacteria biofouling at different velocities was studied. With the increase in flow velocity, the asymptotic value of fouling resistance decreased, while the induction period increased.
[4] The inhibition of advanced materials with Ni-P-rGO coating against iron bacteria biofouling at a different initial concentration was studied. With an increase in the concentration of iron bacteria, the asymptotic value of fouling resistance increased, while the induction period decreased.

## Figures and Tables

**Table 1:** The electroless bath solution composition and operating conditions.

Nickel Sulfate	25 g/L
Sodium Hypophosphite	30 g/L
Citric Acid	15 g/L
Lactic Acid	16 g/L
Sodium Acetate	11 g/L
Potassium Iodide	0.01 g/L
Graphene	40 mg/L
OP-10	Appropriate
pH	4.8
Temperature	83 °C

**Table 2:** Medium composition for iron bacteria.

Ammonium Ferric Citrate	10 g/L
Sodium nitrate	0.5 g/L
Dipotassium phosphate	0.5 g/L
Calcium chloride	0.2 g/L
Magnesium sulfate	0.5 g/L
Ammonium sulfate	0.5 g/L
